# Layer-by-Layer-Coated Cellulose Fibers Enable the Production of Porous, Flame-Retardant, and Lightweight Materials

**DOI:** 10.1021/acsami.3c06652

**Published:** 2023-07-19

**Authors:** Massimo Marcioni, Mengxiao Zhao, Lorenza Maddalena, Torbjörn Pettersson, Roberto Avolio, Rachele Castaldo, Lars Wågberg, Federico Carosio

**Affiliations:** † Dipartimento di Scienza Applicata e Tecnologia, Politecnico di Torino, Alessandria Site, Viale Teresa Michel 5, 15121 Alessandria, Italy; ‡ Institute for Polymers, Composites and Biomaterials, Italian National Research Council, Via Campi Flegrei 34, 80078 Pozzuoli, Naples, Italy; § Department of Fibre and Polymer Technology, KTH Royal Institute of Technology, Teknikringen 56-58, 10044 Stockholm, Sweden

**Keywords:** cellulose, porous materials, layer-by-layer, flame-retardancy, lightweight materials

## Abstract

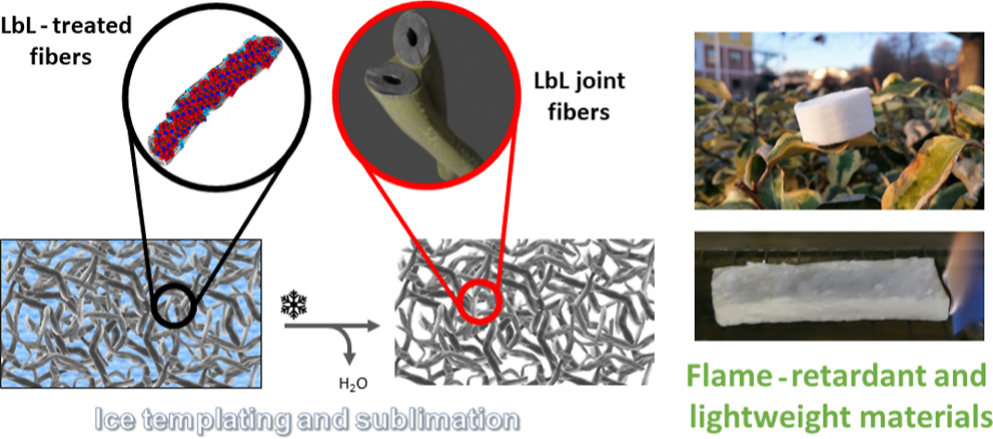

New sustainable materials produced by green processing routes are required in order to meet the concepts of circular economy. The replacement of insulating materials comprising flammable synthetic polymers by bio-based materials represents a potential opportunity to achieve this task. In this paper, low-density and flame-retardant (FR) porous fiber networks are prepared by assembling Layer-by-Layer (LbL)-functionalized cellulose fibers by means of freeze-drying. The LbL coating, encompassing chitosan and sodium hexametaphosphate, enables the formation of a self-sustained porous structure by enhancing fiber–fiber interactions during the freeze-drying process. Fiber networks prepared from 3 Bi-Layer (BL)-coated fibers contain 80% wt of cellulose and can easily self-extinguish the flame during flammability tests in vertical configuration while displaying extremely low combustion rates in forced combustion tests. Smoke release is 1 order of magnitude lower than that of commercially available polyurethane foams. Such high FR efficiency is ascribed to the homogeneity of the deposited assembly, which produces a protective exoskeleton at the air/cellulose interface. The results reported in this paper represent an excellent opportunity for the development of fire-safe materials, encompassing natural components where sustainability and performance are maximized.

## 1 Introduction

In the past decades, the need to find alternative candidates to petroleum-based materials by exploiting a circular economy approach has become more and more important.^1^ The field of insulating materials represents an area of concern. Indeed, current state-of-the-art materials show some sustainability and safety issues related to the use of petroleum-derived solutions with dangerous flammable characteristics.^2^ The development of novel insulating materials encompassing bio-based resources represents an excellent opportunity to solve this problem. Being the most abundant natural polymer, cellulose is one of the main candidates for this task.^3^ However, one of the main challenges when using cellulose in material development is the production of stable, low-density, 3-dimensional (3D) structures similar to polystyrene and polyurethane foams. To this aim, different forms of cellulose ranging from the macroscopic (fibers) to the microscopic (nanocellulose) scale have been investigated in the past years.^4^ In particular, nanocellulose, in the form of either nanofibrils or nanocrystals, has been demonstrated to be a versatile building block for the production of 3D porous structures by exploiting different water-based processing techniques. For example, nanocellulose-based foams can be produced by a surfactant-assisted foaming process.^5^ Alternatively, low-density aerogels can be obtained from CNF hydrocolloids by either freeze-drying^5−7^ or ice templating followed by solvent exchange.^8^ Despite the widespread use of nanocellulose in its many forms, the use of pulp fibers appears to have a more direct practical applicability due to the ease of handling and the reduced costs and environmental impact related to fiber production.^9^ Foaming approaches can also be applied to macroscopic cellulose fibers, as demonstrated by Hutzler et al., who prepared a lightweight cellulose foam using a process aided by sodium dodecyl sulfate.^10^ The production of a stable 3D structure based on cellulose fibers is, however, extremely challenging due to the limited fiber–fiber interactions that might compromise the foam’s mechanical properties and structural integrity. In order to address this problem, Larsson et al. demonstrated the possibility to exploit solvent exchange to obtain cellulose-based lightweight materials starting from self-assembled and cross-linked softwood kraft fibers.^11^ One of the main concerns that is currently limiting the use of cellulose-rich materials in practical applications is related to the extreme flammability of the produced foams.^12^ Unfortunately, the use of conventional FR chemicals, mostly halogen-based, does not represent a solution due to the perceived environmental and toxicological problems potentially arising from their use.^13^ Within this context, phosphate-bearing flame retardants (FRs), with the exception of certain organophosphates,^14^ might represent an environmentally safer alternative.^15^

The production of green, bio-based, and fire-safe cellulose-based materials is thus of significant scientific and societal interest. To this aim, different strategies have been developed over the years.^16^ These mostly include well-established surface modification techniques such as plasma and sol–gel as well as innovative water-based approaches based on either the simple adsorption or self-assembly of nanoparticles and bio-inspired components.^17−20^ A novel and interesting approach to modifying cellulose is represented by the direct assembly of nanoparticles within the swollen fibers.^21^ This strategy could potentially be further developed in order to allow for the bulk inclusion of different FR components. Notwithstanding this, surface modification is still the most exploited method. Within this context, the Layer-by-layer (LbL) assembly technique has been demonstrated to be an easy-to-use and efficient method to impart FR properties to several materials, such as textiles,^22^ polyurethane foams,^23^ and composites.^24,25^ Based on the alternate deposition of positive- and negative-charged components, the LbL assembly leads to the formation of either highly stratified or interpenetrating homogeneous coatings.^26−28^ The versatility of the LbL technique allows it to coat extremely complex 3D structures, such as electro-spun nanofibrous mats.^27,28^ In the field of FR fiber-based materials, some studies have successfully demonstrated how the deposition of a LbL assembly on cellulose fibers prior to their assembly in paper can improve the final mechanical and FR properties.^29−31^ Alternatively, wet-stable lightweight porous fiber networks have been also coated with a FR LbL coating as a post treatment.^32,33^ This latter approach endowed impressive FR properties but required a relatively high number of deposition steps.

**Figure 1:**
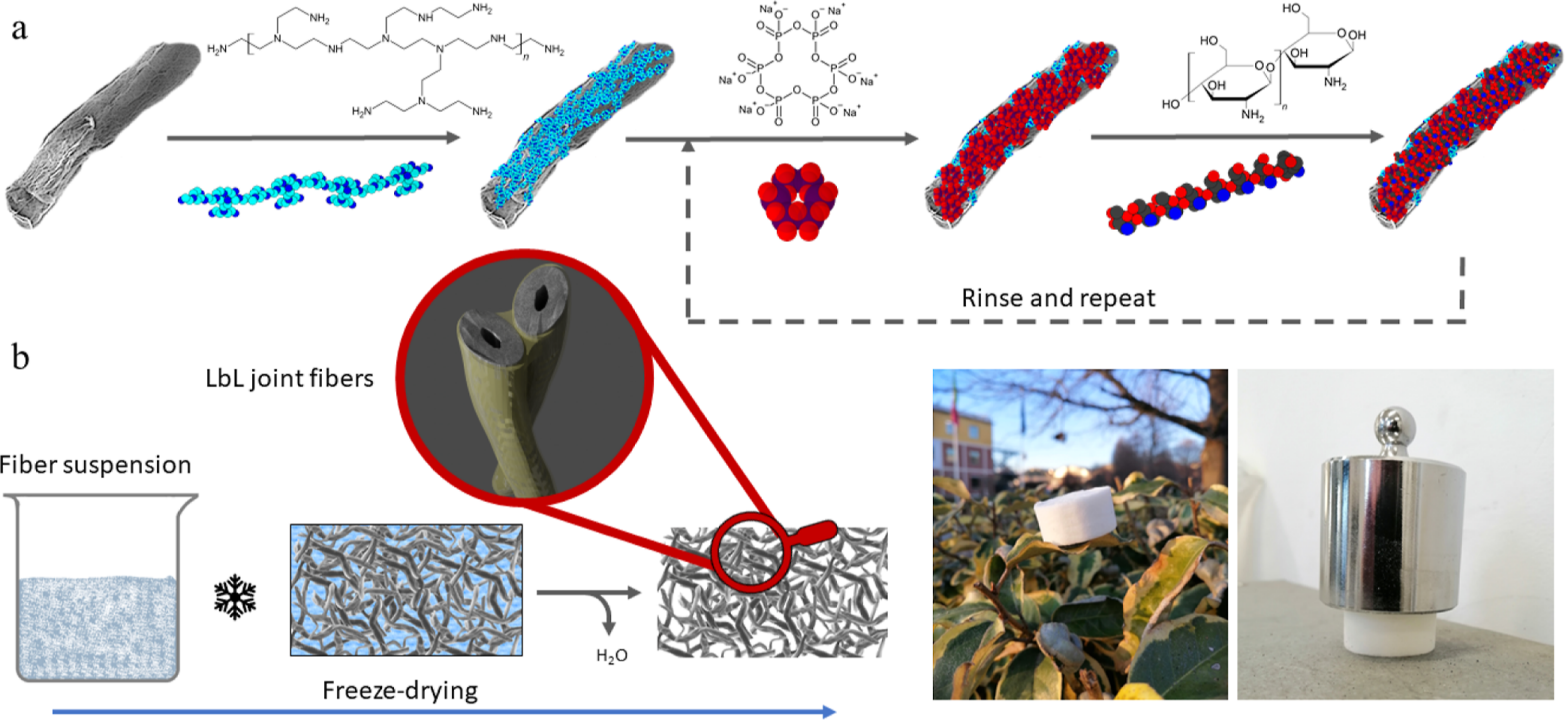
(a) Schematic representation of the deposition of the LbL bilayers onto the cellulose fibers; (b) process used for the porous fiber networks formation and images of the final lightweight material.

In this work, stepping forward from the state of the art, we developed a lightweight cellulose fiber network based on surface-modified cellulose fibers by exploiting an easy and straightforward approach (Figure 1).

The main objective is the production of a green, cellulose-rich, and fire-safe porous material capable of replacing petroleum-based foam applications. The LbL technique is applied to modify the cellulose fiber surface with the aim of including FR properties by using chitosan (CH) and sodium hexametaphosphate (SHMP) as structuring polyelectrolytes. Branched polyethylenimine (BPEI) is used as an anchoring layer due to its ability to improve adhesion and promote the deposition of homogeneous coatings at a low BL number.^34^ CH was selected as a cationic polyelectrolyte due to its good char-forming abilities.^35,36^ SHMP acts as an anionic counterpart and, in combination with CH, produces a FR assembly capable of considerably improving the FR properties of cellulose-based substrates.^37,38^

The presence of the LbL assembly allows for the production of low-density materials by ice-templating followed by sublimation. In the final material, the fiber/fiber interaction is significantly enhanced via the deposited LbL coating, which acts as a glue, joining adjacent fibers with an interpenetrating CH/SHMP assembly. Few deposited bi-layers (BLs), namely, 1, 2, or 3, can actually confer structural integrity. During combustion, SHMP acts as an acid source, favoring the production of thermally stable carbonaceous structures from both cellulose and CH, considerably reducing the release of flammable volatiles, thus resulting in a fire-safe material as evaluated by flammability and forced combustion tests. The approach presented in the present paper, to the best of the knowledge of the authors, has never been reported before and can possibly open up new strategies for the development of advanced materials in many research fields.

## 2 Experimental Section

### 2.1 Materials

Commercial cellulose fibers (ARBOCEL BC1000) with an average length of 700 μm were kindly provided by J. Rettenmaier and Sohne. Branched poly(ethylene imine) (BPEI, *M*_w_ ∼25,000 Da by light scattering, *M*_n_ ∼10,000 by gel permeation chromatography, according to the material datasheet), chitosan (low molecular weight, <20,000 Da and 75–85% deacetylated chitin, according to the material datasheet), acetic acid, and sodium hexametaphosphate (crystalline, +200 mesh, 96%) were purchased from Sigma-Aldrich (Milwaukee, WI). All reagents were used as received for preparing stable water-based solutions by simple solubilization using 18.2 MΩ deionized water supplied by a Q20 Millipore system (Italy). More specifically, chitosan was solubilized in a 1% wt acetic acid aqueous solution and kept under magnetic stirring overnight in order to be completely solubilized. BPEI and SHMP were dissolved in water to 0.1 and 0.5% wt concentrations, respectively, by means of magnetic stirring for 1 h.

### 2.2 Layer-by-Layer Deposition

Prior to LbL deposition, cellulose fibers were rehydrated, and the excess water was removed by centrifugation (Eppendorf centrifuge 5702). Then, fibers were dispersed as 5% wt dispersion in the BPEI solution (10 min) by sonication in a sonication bath (Emmegi C1Livarin) in order to deposit an anchoring layer. The SHMP/CH coating was achieved by alternatively dispersing and sonicating the fibers in the negatively (SHMP) and positively (CH) charged solutions (5 min each). After each deposition step, the excess solution was removed by centrifugation (4400 rpm, 2 min), and fibers were washed (5 min) with water (for BPEI and SHMP) or acetic acid 1% wt (for CH) by a similar dispersion and centrifugation cycle. The process was repeated until a total of 3 BL were deposited (ending with CH in the last and outermost layer) on the fibers. At the end of the LbL deposition, fibers were washed with water to remove any acetic acid remaining from the last washing. Model Si wafer substrates [(100), single side polished] were used to monitor the growth of the selected polyelectrolytes up to 10 BLs by using static dipping following principally the same procedure employed for fibers (layer sequence and deposition times). After each deposition step, the Si wafer was dried in order to collect the IR spectrum of the deposited layer.

### 2.3 Porous Fiber Network Preparation

Wet fibers were dispersed with water to reach a 1:11.5 wt fiber/water ratio and manually shaken to obtain a final fiber-network density of 75 kg/m^3^. The obtained dispersion was poured into molds and frozen at −40 °C for 12 h. Samples were then freeze-dried for 48 h (Toption TOPT-10A, vacuum freeze dryer).

### 2.4 Characterization

The growth of the LbL assembly on silicon wafers [(100), single side polished], for each deposited layer, was monitored using a Fourier transform infrared (FT-IR) spectrophotometer (16 scans and 4 cm^–1^ resolution, Frontier, Perkin Elmer) in the transmission mode. Attenuated total reflectance (ATR) FT-IR spectroscopy spectra for neat and treated cellulose fibers were collected at room temperature in the range 4000–700 cm^–1^ (32 scans and 4 cm^–1^ resolution) using a FT-IR spectrophotometer (Frontier, Perkin Elmer, Italy) equipped with a diamond crystal (with a depth of penetration of 1.66 μm, as stated by the producer). ATR spectra were processed by subtracting the baseline and normalizing by the peak at 1040 cm^–1^.

The surface morphology of untreated and LbL-treated fibers and fiber networks before and after cone calorimetry was studied using a LEO-1450VP Scanning Electron Microscope (imaging beam voltage: 5 kV). Samples were gold sputtered before imaging.

The charge density of BPEI, SHMP, and Chitosan was determined by polyelectrolyte titration using a rapid particle charge titration instrument (Stabino, Meerbusch, Germany) according to a previously described method.^39^ The calculation of the charge density is based on a 1:1 stoichiometric charge ratio between the studied chemicals and the oppositely charged polyelectrolytes. An analytical grade potassium polyvinylsulfate (KPVS) (from Wako Pure Chemicals, Japan) was used to titrate BPEI and CH, while a specially cleaned (ultrafiltration using a tangential flow equipment) and characterized (NMR) polydiallyldimethylammonium chloride (PolyDADMAC), received from Sigma-Aldrich (Stockholm, Sweden), was used for the titration of SHMP.

BPEI, SHMP, and CH were adsorbed onto the cellulose fibers by LbL, as described under 2.2. Filtrates, after deposition of each adsorbed layer, were collected after centrifugation. The concentration of BPEI, SHMP, and CH in the filtrates was determined by titration with KPVS or PolyDADMAC, and the adsorbed mass of the BPEI, SHMP, and CH was back-calculated from the remaining chemicals in the filtrate.

Mechanical properties were evaluated using a dynamometer (Instron 5966, 2 kN cell, Canton, MA) by compressing cylindrical samples with a diameter of 30 mm and a height of 20 mm between two horizontal plates at a constant rate of 5 mm/min. The compressive modulus was calculated from the initial linear section of the compression curves between 2 and 6% compression strain. Prior to the tests, samples were conditioned at 23.0 ± 0.1 °C for 48 h at 50.0% ± 0.1 RH in a climatic chamber.

The flammability of the prepared samples was evaluated in horizontal and vertical configurations; the sample (80 × 25 × 10 mm^3^) was ignited from its short side by a 20 mm methane flame (flame application time: 2 × 6 s). The test was repeated at least 2 times for each formulation; during the test, parameters such as after-flame time (the time for which flame persists after the ignition source has been removed on the basis of ISO 13943, expressed in s), char length (mm), and final residue (%) are used.

Cone calorimetry (GA01, ISO 5660, Noselab ats, Italy) was employed to investigate the combustion behavior of square samples (50 × 50 × 15 mm^3^) under exposure to a heat of 35 kW/m^2^ in horizontal configuration following an earlier described procedure.^40^ The following parameters were registered: time to ignition (TTI, s), average and peak heat release rates (HRR and pkHRR, kW/m^2^), total heat release (THR, MJ/m^2^), and final residue (%). The test was repeated 3 times for each formulation in order to ensure reproducibility; the experimental error was assessed as the standard deviation (σ).

Raman spectra of the sample after cone calorimetry were obtained on an InVia Raman Microscope (Renishaw, New Mills, UK, argon laser source 514 nm) coupled with a Leica DM 2500 optical microscope. The D and G bands were fitted with Lorentz functions in order to determine their ratio.

## 3 Results and Discussion

### 3.1 Coating Growth and Assembly on Fibers

**Figure 2:**
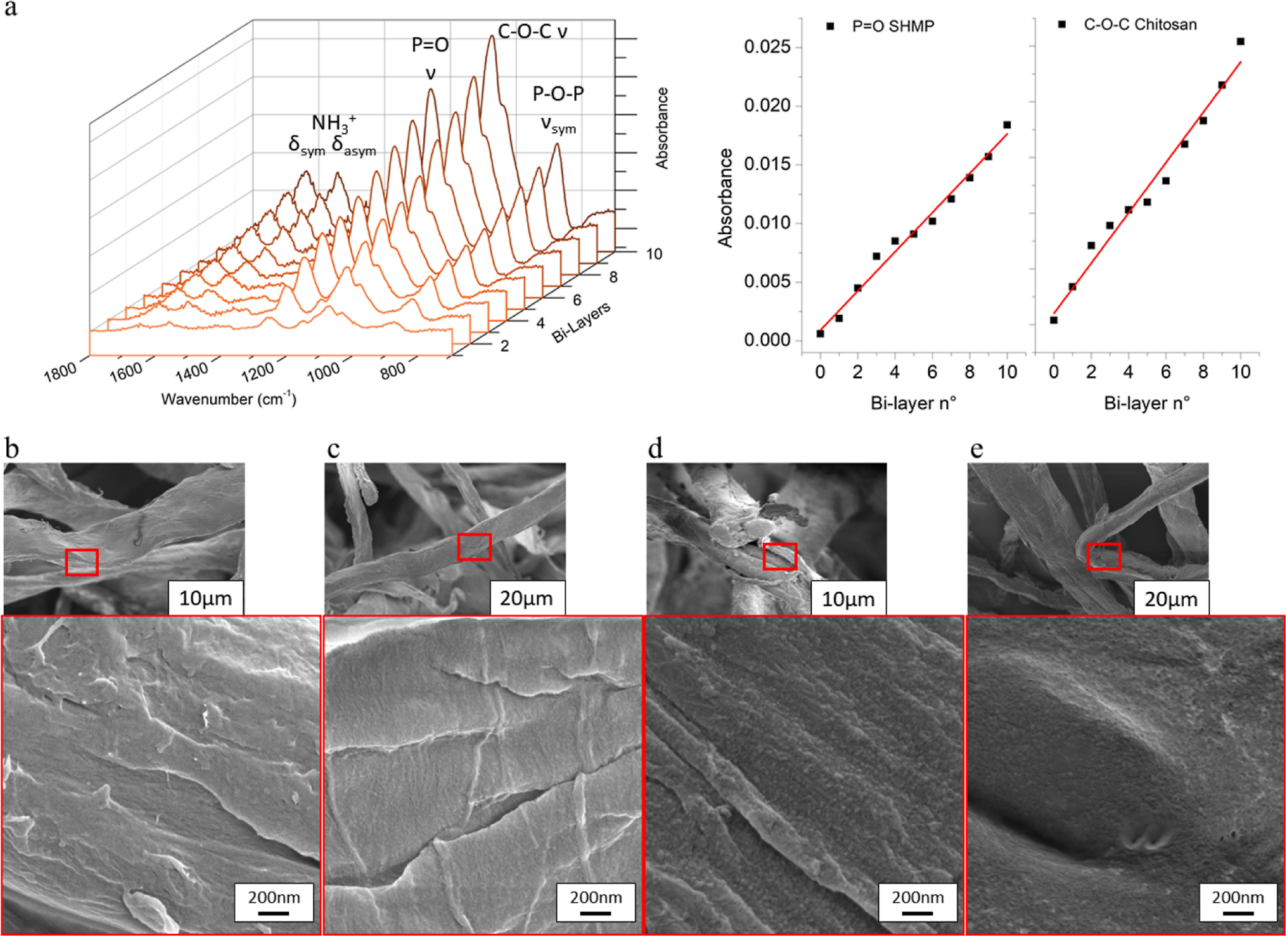
(a) FT-IR spectra showing the LbL growth on a silicon wafer substrate; SEM images of (b) neat cellulose fibers and (c) 1 BL-, (d) 2 BL-, and (e) 3 BL-coated fibers.

The build-up of the LbLs, up to 10 BLs, was first studied on a model silicon substrate with the aid of transmission IR spectroscopy.^41^ BPEI is employed as a starting layer to improve the adhesion between substrate and assembly. The spectra of pure substances, BPEI, CH, and SHMP, deposited by drop casting on Si wafers, were also evaluated as a reference (Figure S1). CH shows a broad band between 3700 and 3000 cm^–1^ related to water −OH group stretching vibration overlapping the stretching vibration of amine groups typically found in the 3550–3330 cm^–1^ region.^42^ A water bending signal is also found at 1643 cm^–1^ and the C–H bond in CH_2_ and CH_3_ are visible at 2900 and 2880 cm^–1^, respectively. The CH most intense signal is located at 1074 cm^–1^, and it is related to the stretching vibrations of the C–O–C group.^43^ The N–H absorption of amines and protonated amines is found in the 1650–1500 cm^–1^ range, and the latter produce two bands at 1643 and 1555 cm^–1^ related to the asymmetric and symmetric bending vibrations, respectively.^40,42^ SHMP characteristic peaks attributed to −PO_4_ groups can be found in the finger-print region of the spectra. The peak at 1260 cm^–1^ is related to the stretching of P=O, while at 876 cm^–1^, it is possible to find the peak related to P–O–P symmetrical stretching.^22,42^Figure 2a shows the spectra collected during the build-up of the LbLs on silicon wafers up to 10 BL and the intensity vs BL number plot for the characteristic peaks of CH and SHMP. The steady increase in signal intensity as the number of deposited BL increases indeed shows the successful LbL assembly of the selected components. The absorbance at 1260 cm^–1^ and 1074 cm^–1^, corresponding to the P=O group of SHMP and C–O–C stretching of chitosan, respectively, plotted as a function of the deposited BL number, suggests a linear regime growth.^37^ The BPEI(SHMP/CH)_*n*_ assembly was then transferred to cellulose fibers by depositing *n* = 1, 2, or 3 BLs, aiming for a short and efficient deposition process. Figure 2b–e shows a collection of scanning electron microscopy (SEM) micrographs of uncoated and LbL-coated fibers. Neat cellulose shows the typical corrugated morphology of natural fibers with a well-visible surface texture. The deposition of the first two BLs on the fibers appears to be very thin and barely visible in SEM images (Figure 2c,d). However, for 3 BLs, the coating appears more evident by displaying a homogeneous coverage on all the fibers (Figure 2e).

**Figure 3:**
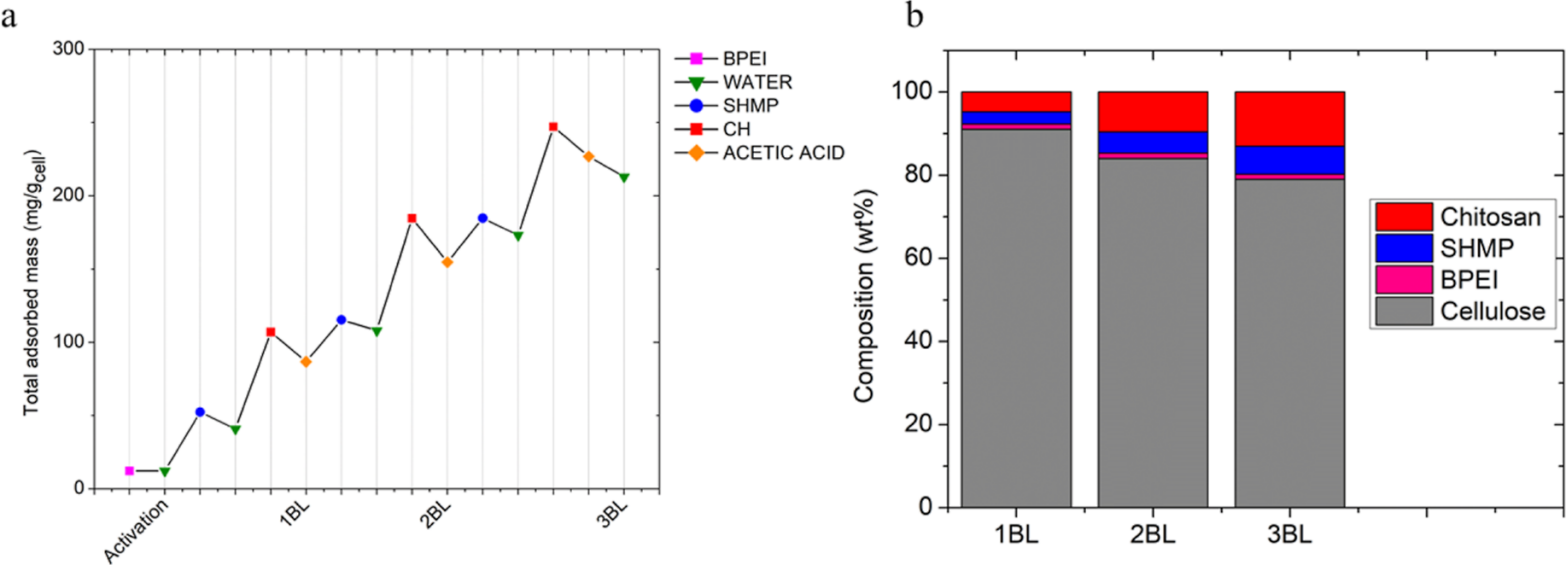
(a) Total adsorbed mass for cellulose-rich fibers as evaluated with charge titration for each individual adsorption step, including rinsing during the build-up of 3 BL; (b) composition of dry fibers in % wt, calculated from the charge titration measurement.

The development of a LbL assembly on the fibers was further evaluated by ATR-IR spectroscopy (Figure S2). While it is difficult to discriminate CH signals as they overlap with signals of cellulose, the presence of a P=O band, ascribed to SHMP, is clearly visible. This band increases by increasing the number of deposited BLs, thus supporting the formation of a LbL assembly. Polyelectrolyte titration was employed to determine the amount of each polyelectrolyte assembled on the fibers, which was back-calculated from the polyelectrolyte titration of the non-adsorbed components after centrifugation and is shown in Figure 3a. The charge densities of SHMP and CH were found to be 11.31 and 6.31 meq/g, respectively, resulting in an estimated charge neutralization complexation ratio of 1:2 (w/w). With these charge titrations, it is possible to better characterize the assembly onto the fibers, showing that the linear growth observed on the silicon wafer was maintained also for the cellulose fibers. The results show that the totally adsorbed mass increases steadily for each layer, followed by a slight decrease in the rinsing step, most probably due to the desorption of loosely bound excess polyelectrolytes during the rinsing. When evaluating the coating composition, as demonstrated in Figure 3b, it can be detected that CH is the main component in the coating, with values ranging from 53 to 62%. This is expected and attributed to charge density compensation phenomena according to the estimated charge complexation ratio between CH and SHMP. Once deposited, the coating accounts for 9, 16, and 21% for 1, 2, and 3 BL of the total mass of the composite, respectively.

### 3.2 Low-Density Fiber Network Forming and Characterization

**Figure 4:**
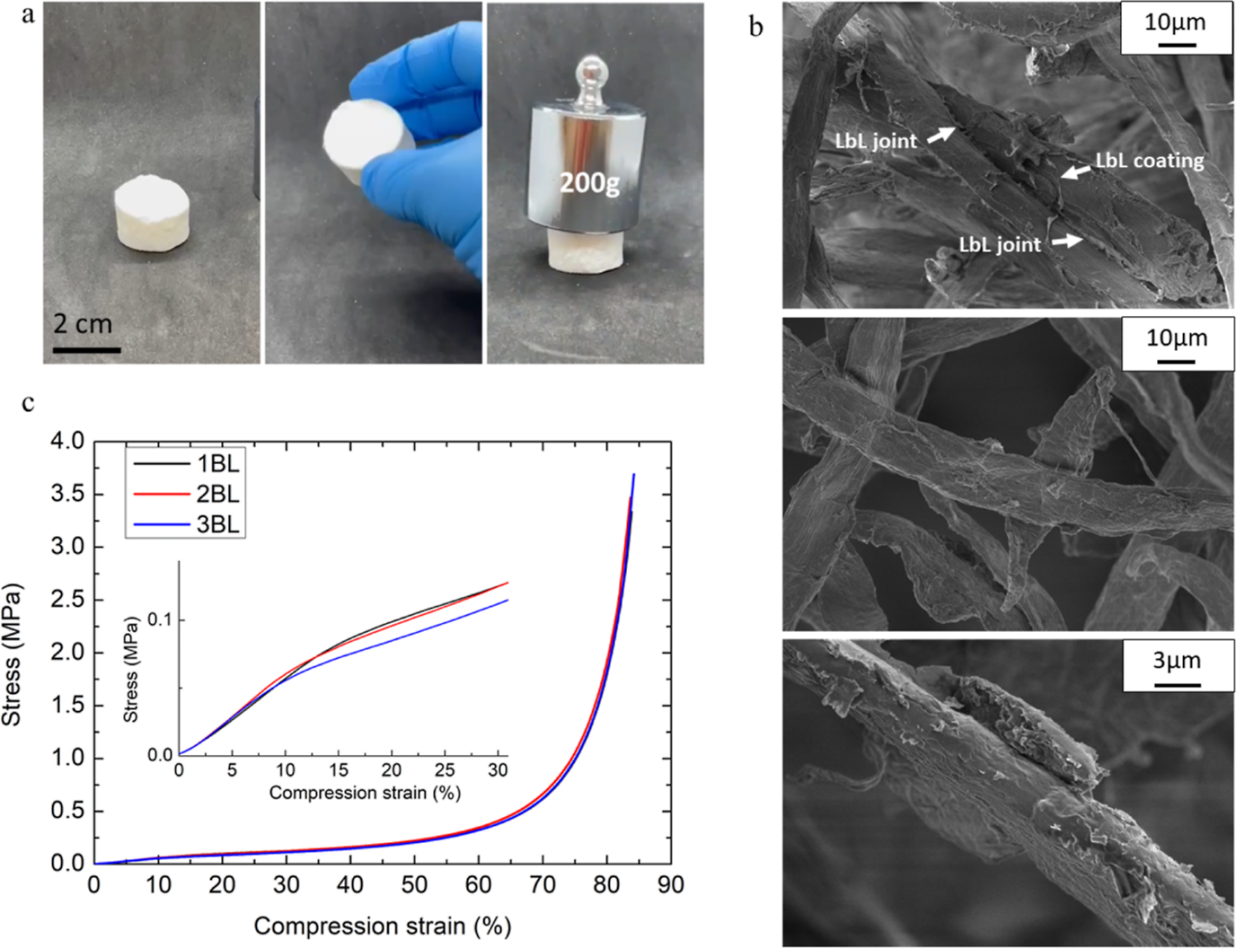
Fiber network morphological and mechanical characterization: (a) digital images of the as-prepared fiber networks, (b) SEM images at different magnifications of a 3 BL sample in which the coating interconnection is visible between fibers, and (c) compression stress/strain plots for 1, 2, and 3 BL samples with an insert showing a higher magnification of the compression range 0–30%.

Self-standing porous fiber networks were prepared by freeze-drying the suspensions of LbL-coated fibers. Figure 4 shows digital images of the material prepared from 3 BL-coated fibers, SEM images at different magnifications, and stress/strain plots from compression tests.

As reported in Figure 4a, the freeze-drying procedure produces porous fiber networks that can be easily handled and can bear the compression of a static weight (100 times its weight). Upon investigation of the fiber-network microstructure, it is possible to observe how fibers are randomly arranged in space, producing a 3D structure whose structural integrity is mainly due to the physical interaction between the fibers. Higher magnifications reveal that bridges exist between different fibers due to the interlocking of the LbL coating as the fibers are pushed together by the ice crystal formation during the freezing procedure since the fibers will “escape” the crystals formed during the freezing process. It is worth mentioning that such stable 3D structures cannot be produced from the untreated fibers, where the freeze-drying resulted in an extremely soft and collapsed structure that broke upon removal from the silicone mold (Figure S3). This suggests that the coating asserts a major role in maintaining the 3D structure of the final network while producing an exoskeleton at the air/cellulose interface. The presence of the film-forming joints is more visible as the number of BLs increases, and for 3 BLs, the bridges are much easier to detect due to the higher amount of adsorbed coating (Figure 4b). By contrast, such bridges and joints were not detected in the freeze-dried dispersions of uncoated fibers (Figure S3). As far as mechanical properties are concerned, upon compression, the samples show progressive collapsing of the structure, eventually reaching a densification stage at high deformations, which is typical of rigid foams.^44^ Interestingly, the measured stress/strain plots for samples prepared with fibers coated at 1, 2, and 3 BLs are almost superimposed, suggesting that the compression strength and modulus are not correlated to the amount of coating on the fibers at such a low number of BLs (Figure 4b). The average compressive modulus is found to be 0.60 ± 0.03 MPa. Although the production of a reference material with uncoated fibers was not feasible, it is possible to compare the achieved results with similar porous fiber networks prepared from self-assembled and cross-linked cellulose fibers.^11^ These latter show compressive moduli ranging from 0.6 to 1.1 MPa depending on the density. Other cellulose-based foams produced with cellulose nanofibrils achieved higher compressive moduli, as expected.^45^ This suggests that the materials developed in this paper could easily be handled and employed as a lightweight panel where no load-bearing functions are required.

### 3.3 Thermal Stability

**Figure 5:**
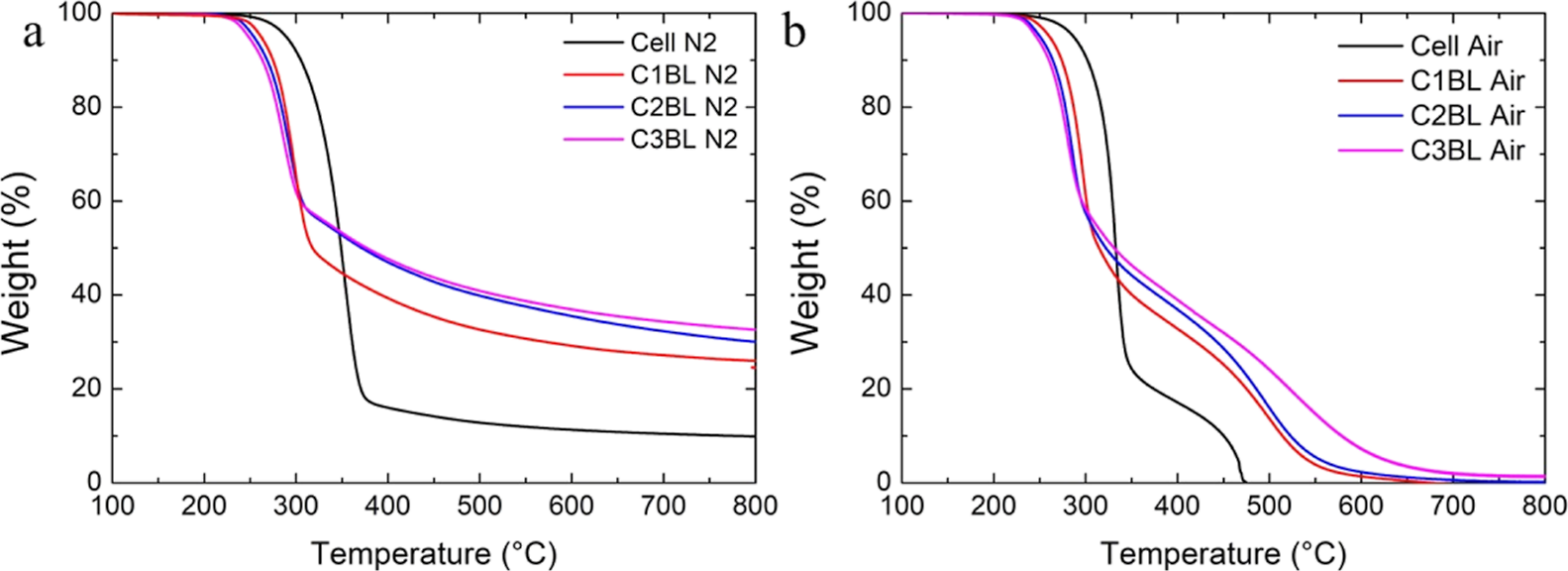
TG plots of neat cellulose and 1, 2, and 3 BL fiber networks in (a) nitrogen and (b) air.

Thermogravimetric analyses in nitrogen and air were used to investigate the thermal and thermo-oxidative stability of the prepared fiber networks. The aim is to obtain fundamental information on the effect of the deposited coating on the fiber char-forming abilities. The resulting TG plots are reported in Figure 5 and the data in Table S1.

In an inert atmosphere, cellulose shows its well-known single decomposition step resulting from the depolymerization of the glycosyl units to volatile products (such as levoglucosan) in competition with the dehydration of the same units to give a thermally stable char accounting for 10% of the initial mass.^46,47^ The LbL-treated cellulose fiber exhibits an early decomposition (*T*_on 5%_ of 289 and 260 °C for neat cellulose and coated fibers, respectively), which is generally reported for phosphate-containing systems and is considered positive since it favors the cellulose decomposition toward char formation^48^ and results in the release of a reduced amount of volatile combustible products.^22^ The final residues at 800 °C increase significantly up to 33% for 3 BL-coated fibers, indicating a substantial improvement in cellulose char formation. Interestingly, the amount of residue collected at the end of the test is not proportional to the deposited BL number (Table S1). This suggests that increasing the number of deposited BL produces diminishing results in char formation during TG tests. Such behavior, already observed on other cellulose-based substrates,^37^ points out that a good char-forming efficiency of the deposited CH/SHMP assembly can be achieved even at low BL numbers. In air, Figure 5b, cellulose undergoes a two-step thermal oxidation path. The first step (between 250 and 350 °C) is attributed to the production of both volatiles and aliphatic char. This latter is further oxidized during the second step (between 350 and 480 °C), producing CO and CO_2_ and leaving a negligible residue.^33^ In an oxidative environment, the presence of the LbL coating also produces an anticipation in decomposition and a conspicuous increase in char production during the first weight loss step (residual weight at 400 °C is nearly doubled for LbL-coated fibers). Such residues appear to be more stable as their oxidation during the second step occurs gradually and persists up to higher temperatures with respect to neat cellulose. Indeed, 3 BL-coated fibers still show a residual 10% weight up to 600 °C, thus evidencing how the presence of the coating can simultaneously improve the amount and quality of the produced char. This occurs as a consequence of the double effect exerted by SHMP, which can directly improve cellulose char formation while also building up a protective insulating layer with CH, which further improves char production due to a heat shielding effect.^37^

### 3.4 Flammability and Combustion Properties

**Figure 6:**
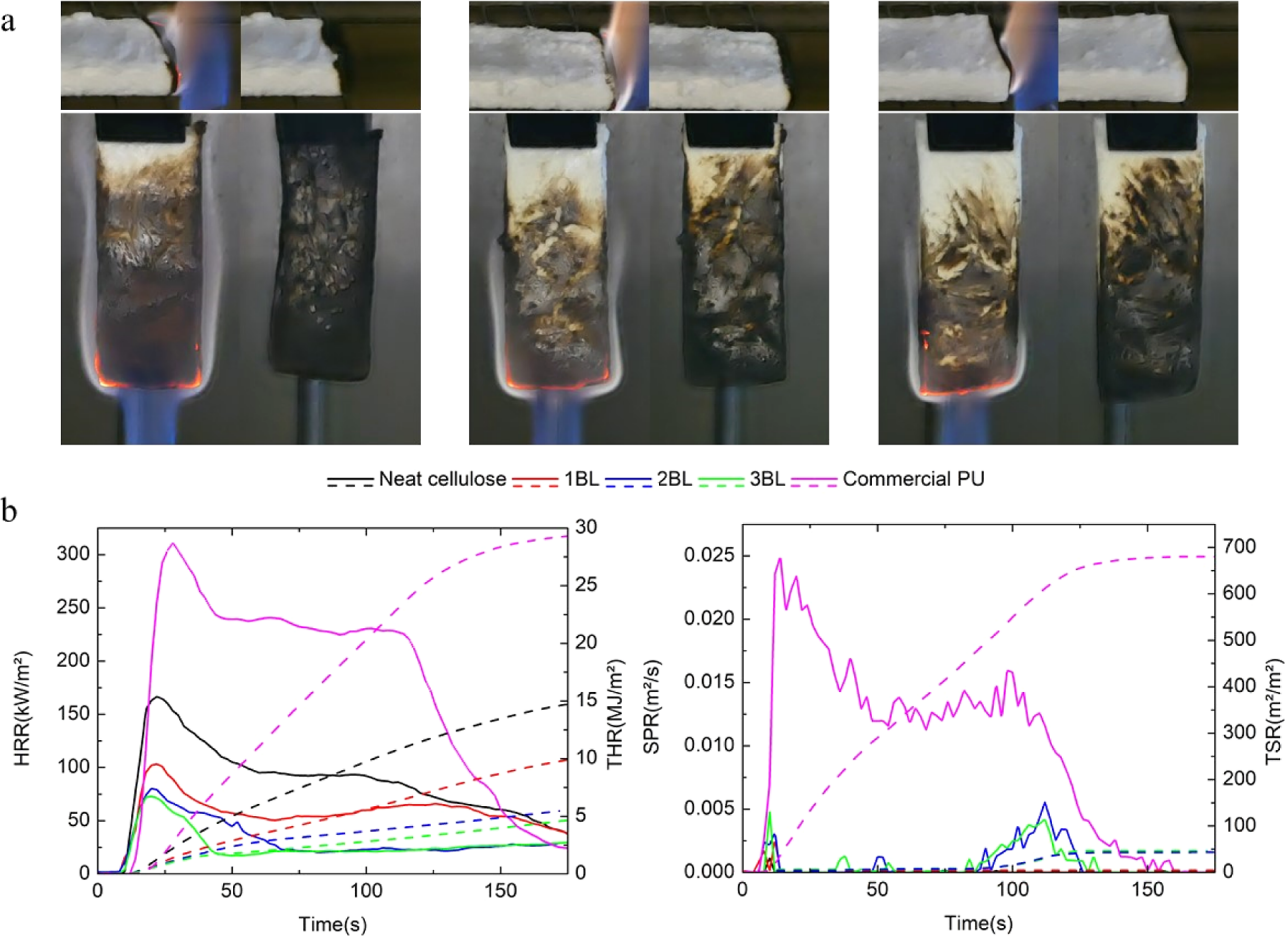
Flammability and cone calorimetry characterizations: (a) snapshot from horizontal and vertical flame tests for 1 BL, 2 BL, and 3 BL fiber networks (from left to right) with the flame and after the flame was removed. Cone calorimetry results of cellulose fiber networks (b) HRR and THR on the right and SPR and TSR on the right.

Flammability tests and forced combustion tests by cone calorimetry have been performed in order to evaluate the FR properties of the prepared fiber networks. This set of characterizations provides complementary information on the simulated behavior of a specimen during a fire. Indeed, by evaluating the reaction of the sample to exposure to a direct flame, flammability tests assess the propensity of the material to start a fire.^49^ Conversely, by exposing the sample to a heat flux typically found in a developing fire, forced combustion tests evaluate the fire spreading potential of the material while also allowing for a preliminary evaluation of the amount of smoke produced during combustion.^50^Figure 6 collects digital images of the samples during flammability tests and cone calorimetry plots associated with heat release rate (HRR) and smoke production rate (SPR), as well as their integral values. A commercially available rigid polyurethane foam (PU) was also evaluated as a comparison for flammability and cone calorimetry tests due to the widespread use of this kind of foam in many industrial products and applications.^51^Tables S2 and S3 collect the data from the flammability and cone calorimetry test for all tested samples, respectively.

**Figure 7:**
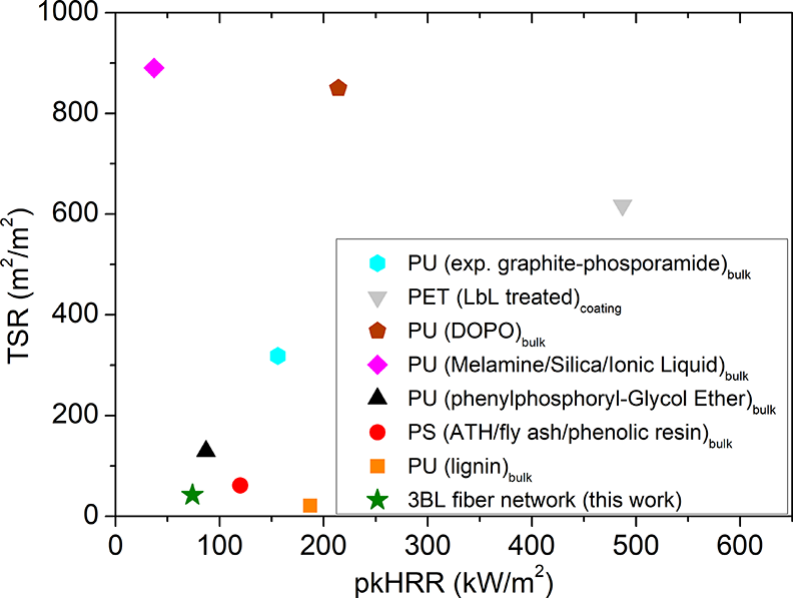
Comparison of cone calorimetry results of different rigid FR foams from the literature background. PET: polyethylene terephthalate, PS: polystyrene, DOPO: 9, 10-dihydro-9-oxa-10-phosphaphenanthrene-10-oxide, and ATH: aluminumtrihydroxide.

Flammability in horizontal configuration was first evaluated as this is typically adopted for foamed materials. Untreated cellulose fibers, compacted in the same shape as samples of fiber networks, have been used as reference materials. Upon exposure to the methane flame, the neat cellulose fibers quickly ignite and undergo complete combustion with vigorous flames. Conversely, when the flame is applied to 1 BL, after ignition, the flame is not able to completely burn the sample, and it rapidly extinguishes, leaving the majority of the sample intact. 2 and 3 BL samples further improve the 1 BL performances by self-extinguishing the flame immediately after removal of the methane flame. Flammability in vertical configuration was also tested. The vertical setup represents a harsher condition compared to the horizontal one and is normally considered a key test for evaluating the fire safety of dense materials such as polymer composites and nanocomposites. The 1 BL sample burned completely after the methane flame removal, forming a stable carbon structure. Surprisingly, the 2 BL samples showed a self-extinguishing behavior where, after ignition, the flame extinguished after a few seconds, leaving the structure of the sample almost intact. The best results were obtained by 3 BL samples, which self-extinguished the flame immediately after the methane flame was removed. Such results clearly point out the extreme fire safety achieved by these fiber networks, especially if compared with the reference commercial PU foams that burned completely during both horizontal and vertical flame tests (Figure S3). Forced combustion tests were also performed to evaluate the reaction of the samples when exposed to an irradiating heat flux of 35 kW/m^2^, which is a value typically found in developing fires. As for flammability tests, neat cellulose fibers were employed as a reference sample. Commonly, upon exposure to the heat flux, the sample starts decomposing and releases flammable volatiles that ignite when the proper concentration is reached, leading to flaming combustion. The neat cellulose fibers quickly ignite and burn with vigorous flames, reaching a pkHRR of 167 kW/m^2^ while almost being totally consumed by the combustion (final residue <1%). The presence of the LbL coating considerably changes the burning behavior of the fibers assembled in the porous networks. Indeed, during combustion, extensive charring of the samples occurs, allowing the samples to maintain their original shape and structure (Figure S4). This leads to conspicuous reductions in both pkHRR and THR values. A single BL produces a 38% reduction in pkHRR and 31% in THR, thus showing the great FR potential of this system. The best performances were obtained with 3 BL with a pkHRR and THR reduction of 56 and 68%, respectively. It is worth highlighting that 2 and 3 BLs samples achieve extremely low HRR values (below 100 kW/m^2^), which are well below the reference PU foam and similar to high-performing foams, such as phenolic resins.^52^ These excellent results can be ascribed to the char-forming properties of the SHMP/CH pair that, during combustion, can produce thermally stable carbonaceous structures, which protect the fiber underneath by limiting heat and mass transfers. In addition, the presence of SHMP and the protective structures further limit the release of combustible volatiles from the sample by favoring cellulose char formation. A single BL is therefore enough to stop flame spread in horizontal configuration since, after ignition, the release of flammable volatiles is not enough to sustain combustion, leading to a self-extinguishing behavior even after multiple flame applications (Video S1). Conversely, 2 and 3 BLs are required to achieve the same results in vertical configuration. By cone calorimetry, the abovementioned phenomena considerably hinder the combustion rates of the samples, as shown by the strong reduction in pkHRR and THR values. Such inefficient combustion inevitably produces an increase in total smoke release (TSR) values with respect to neat cellulose. However, it is worth mentioning that TSR values commonly measured for synthetic foams are typically 1 order of magnitude higher than those measured for these LbL-stabilized fiber networks, as shown by the results achieved by the employed rigid PU reference (679.4 vs 42.2 m^2^/m^2^ for the PU and the 3 BL sample, respectively). Since smoke is often considered one of the main causes associated with fire casualties due to both its incapacitating effects that prevent people from escaping and the produced long-term damages,^53^ the relatively low TSR values measured clearly show the potentialities of the prepared fiber networks in replacing commercial petroleum-based foams. This fact can be further highlighted by performing a comparison of pkHRR and TSR values from cone calorimetry tests of previously reported FR rigid foams (Figure 7).^54−60^

The performed comparison clearly highlights how the extremely limited pkHRR and TSR values of the porous fiber networks prepared in this article actually outperform the values achieved by widely employed rigid foams such as PS, PET, and PU.

### 3.5 Post Combustion Residue Analysis

**Figure 8:**
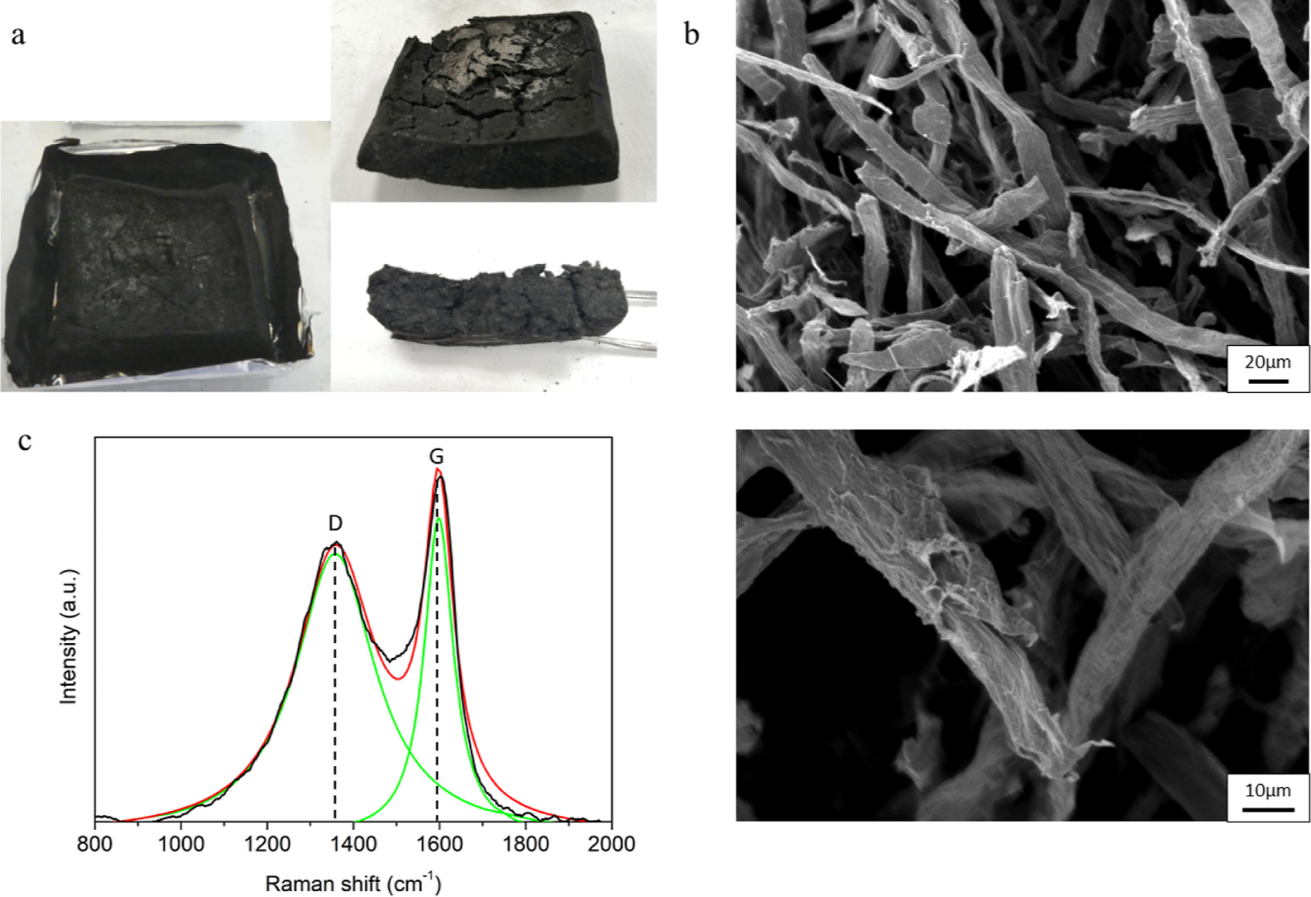
Residue analysis for 3 BL-coated fiber networks after cone calorimetry test: (a) digital images of the residues, (b) SEM images at different magnifications and (c) Raman spectrum with fitted peaks.

The combustion residues collected after cone calorimetry tests were characterized by means of SEM and Raman. Figure 8 collects the digital images of the 3 BL residue, SEM observations at different magnifications, and the Raman spectrum with deconvoluted signals for the 3 BL fiber network. Digital images of the cone calorimetry residue for neat cellulose and 1 and 2 BLs are reported in Figure S4.

After combustion, the fiber networks prepared from coated cellulose produced a self-sustaining residue, which can be easily handled. This is particularly apparent for the 2 and 3 BL samples. SEM investigations show a structure that resembles that of the unburned fiber network. The coating becomes more wrinkled due to the char formation processes that occurred during combustion; this is visible also for 1 and 2 BL fiber networks (Figure S6).

**Figure 9:**
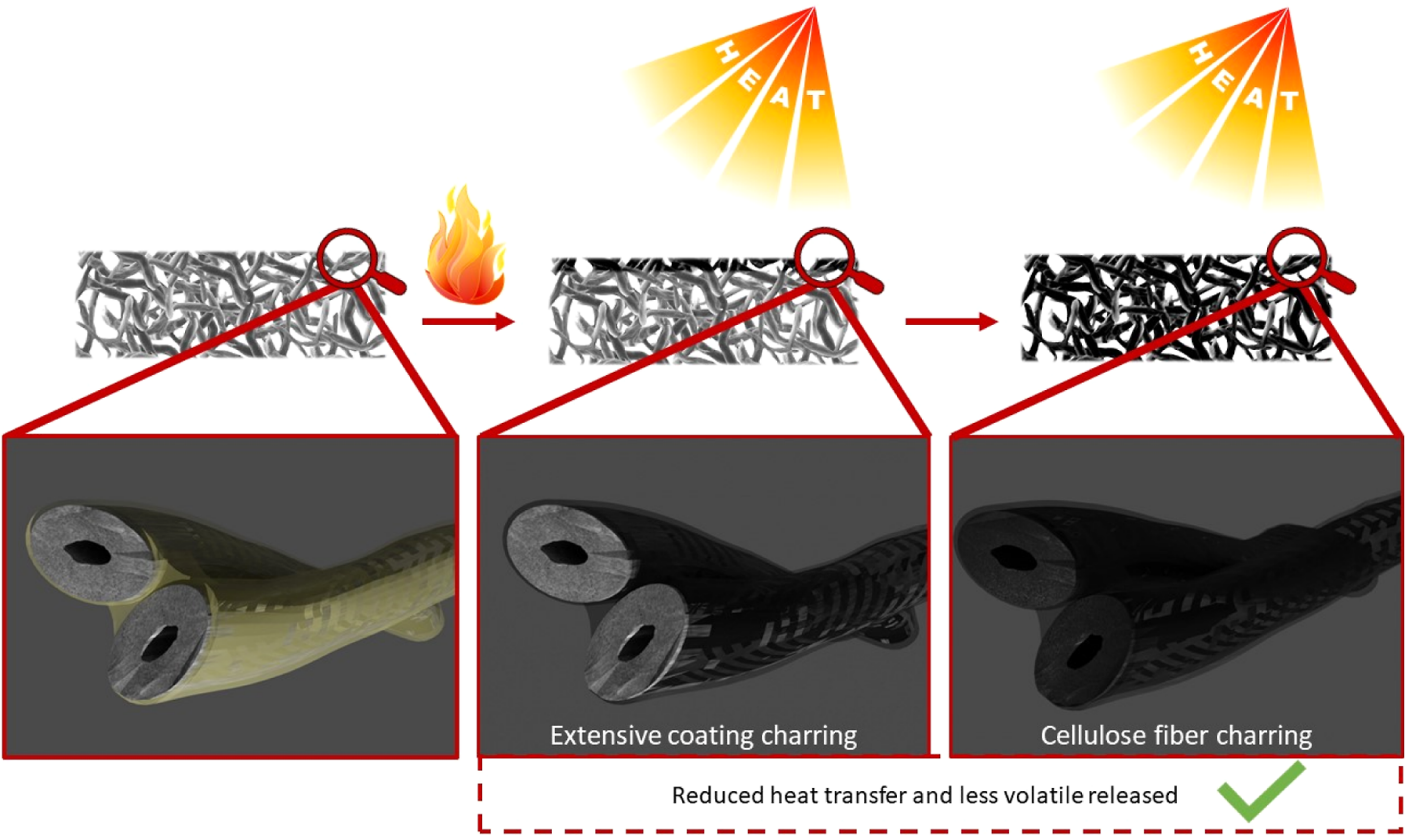
Schematic representation of the fiber network FR mechanism upon exposure to a flame or heat flux.

Raman spectroscopy points out the formation of two characteristic signals, known as the G and D bands, normally found in graphene and graphene-related materials.^61^ The G-band is linked to the stretching of sp^2^ carbons and is the primary mode in graphene and graphite. The D-band is known as the disorder- or defect-band and is normally associated with the presence of edges, curvature, and inclusions close to sp^2^ carbon rings. For residues produced from cellulose pyrolysis, these latter two bands are often broadened due to the contribution of several additional peaks associated with the complexity of the structures produced upon thermal decomposition.^62^ This is also observed on the D band of the Raman spectrum of the shrunken and brittle residue collected from neat cellulose after cone calorimetry (Figure S7). Conversely, the presence of well-defined G and D bands suggests an improved graphitization degree of the cellulose network (Figures 8 and S8). This is further supported by the evaluation of the ratio between the area underneath the G and D bands, which is often employed to evaluate the quality of the produced char structure. The collected signals present D/G values ranging from 2.2 to 2.4 that are similar to those already reported for graphene oxide-containing combustion residues.^43^ 1 and 2 BL fiber networks show similar results (Figure S8). This further confirms the crucial role of the LbL coating in promoting cellulose char to form decomposition pathways that considerably reduce the production of combustible volatiles. Based on the performed characterization, it is possible to preliminary devise a FR mechanism for these LbL-enabled fiber networks (Figure 9).

Upon exposure to a flame or a heat flux, CH undergoes extensive charring thanks to the presence of SHMP.^33^ The SHMP also favors the dehydration of cellulose glycosyl units toward the production of a thermally stable char. The coating charring helps in maintaining the fiber network’s structural integrity, thus resulting in reduced heating rates that also promote cellulose char production from the bulk.^47^ The overall process considerably limits the amount of volatiles feeding the flame, thus producing the observed self-extinguishing behavior in flammability tests and low heat release rates during cone calorimetry. Such high FR efficiency can thus be ascribed to the homogeneity of the deposited CH/SHMP assembly, which can exert a considerable FR effect even at such low BL numbers by producing a continuous exoskeleton at the air/cellulose interface.

## 4 Conclusions

Low-density and FR porous fiber networks have been prepared from LbL-functionalized cellulose fibers by means of an easy and straightforward approach. Chitosan and sodium hexametaphosphate were selected as coating constituents. Their LbL assembly was studied on model Si surfaces by FT-IR and on cellulose fibers by polyelectrolyte titration, pointing out a linear growth regime. Fibers coated by 1, 2, or 3 BLs were employed for the production of porous fiber networks by means of a freeze-drying approach. The presence of the coating was found to be the key factor, enabling the formation of a self-sustained porous structure by enhancing fiber–fiber interactions during ice-templating. The prepared fiber networks displayed a 3D structure with an open porosity where cellulose fibers are interconnected by joints produced between the LbL assemblies that also form an exoskeleton at the air/cellulose interface. The cellulose fiber content is extremely high and ranges from 90 to 80% wt for fiber networks produced from 1- or 3 BL-coated fibers, respectively. The FR characterization performed by flammability and cone calorimetry tests pointed out how these fiber networks can easily self-extinguish the flame in vertical configuration while also displaying extremely low combustion rates (HRR below 100 kW/m^2^) and smoke release (1 order of magnitude lower than commercially available PU foams). Such results are comparable to other high-performing FR solutions based on synthetic polymers and become even more impressive when considering the extremely high cellulose fiber content. These results show that a minimum of 3 BLs of the selected assembly are required to confer optimal FR properties in both flammability and cone calorimetry testing. Further developments might involve a change in coating composition aimed at targeting additional FR properties or improved mechanical resistance. In conclusion, the proposed approach can pave the way to the development of green and sustainable high-performing materials, where the final properties can be easily tuned by exploiting the versatility of the LbL approach in order to find the required balance between performance and sustainability.

## Supporting Information Available

IR spectra of pure substances, ATR-IR spectra of coated fibers, images of polyurethane foam flammability, flammability data, TGA data, cone calorimetry data, digital photos, SEM images, and Raman spectra of cone calorimetry residues (PDF)
Flammability test in horizontal configuration of porous fiber networks prepared from 1BL coated fibers (MP4)
