# Supplementary material for: Layer-by-Layer-Coated Cellulose Fibers Enable the Production of Porous, Flame-Retardant, and Lightweight Materials
Source: ACS Appl Mater Interfaces. 2023 Jul 19;15(30):36811–21. doi: 10.1021/acsami.3c06652 (PMC10401563; doi:10.1021/acsami.3c06652)
Supplement: Supplementary file 1 — am3c06652_si_001.pdf [file am3c06652_si_001.pdf]

SUPPORTING INFORMATION

Layer-by-Layer Coated Cellulose Fibers Enable the Production of Porous, Flame Retardant and Lightweight Materials

Massimo Marcioni,[a] Mengxiao Zhao,[c] Lorenza Maddalena,[a] Torbjörn Pettersson,[c] Roberto Avolio,[b] Rachele Castaldo,[b] Lars Wågberg,[c] Federico Carosio,*[a]

[a] Dipartimento di Scienza Applicata e Tecnologia, Politecnico di Torino, Alessandria Site, Viale Teresa Michel 5, 15121, Alessandria, Italy

[b] Institute for Polymers, Composites and Biomaterials, Italian National Research Council -Via Campi Flegrei 34, 80078 Pozzuoli, NA, Italy

[c] Department of Fibre and Polymer Technology, KTH Royal Institute of Technology, Teknikringen 56-58, 10044 Stockholm, Sweden.

Corresponding author: federico.carosio@polito.it

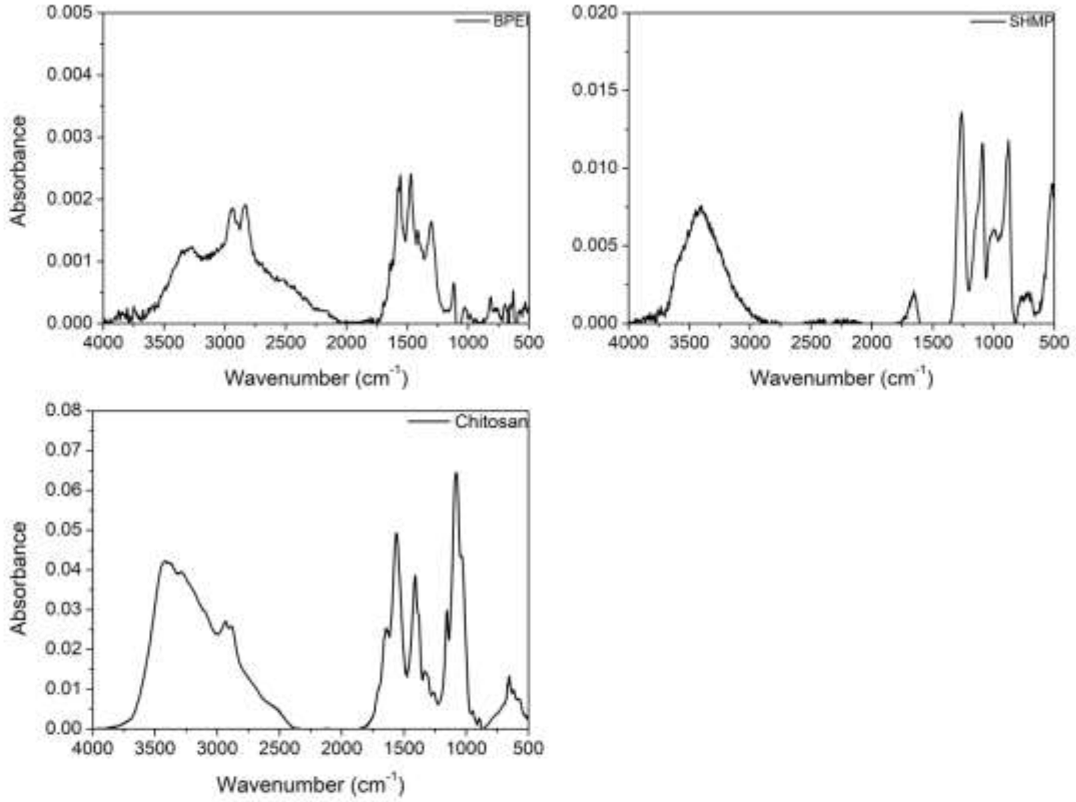

Figure S1. FT-IR spectra of the individual chemicals used in LbL deposition.

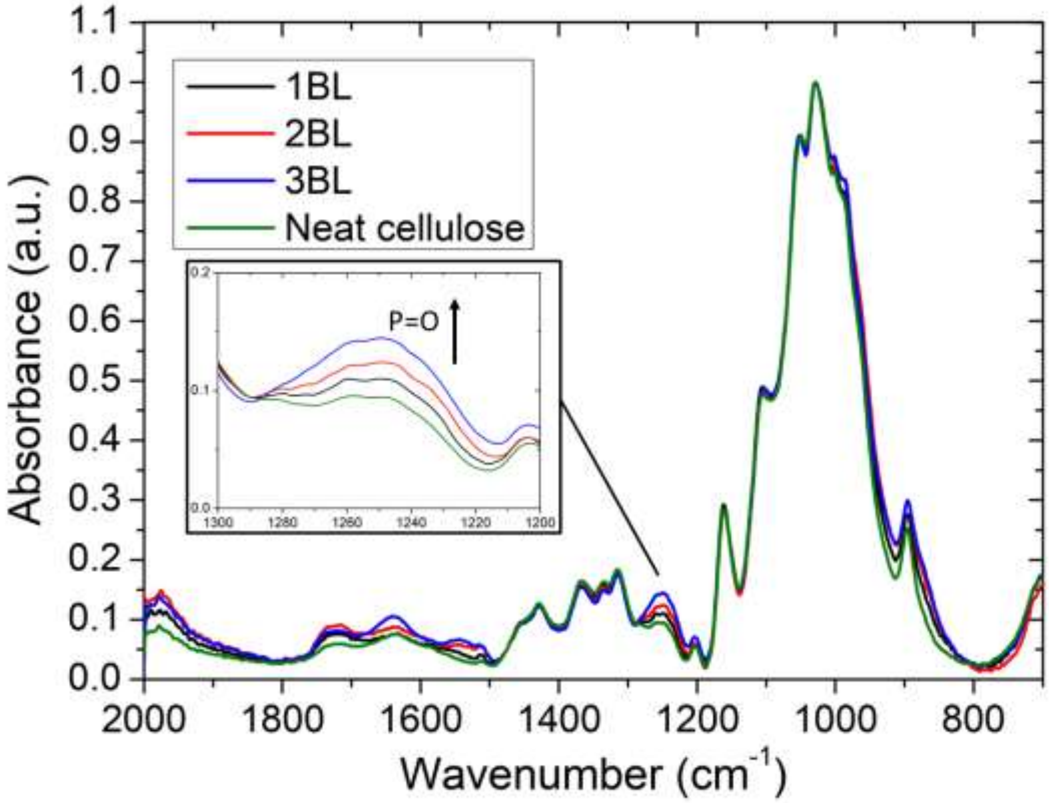

Figure S2. ATR-IR of 1,2 and 3BL coated cellulose fibers vs neat un-coated cellulose.

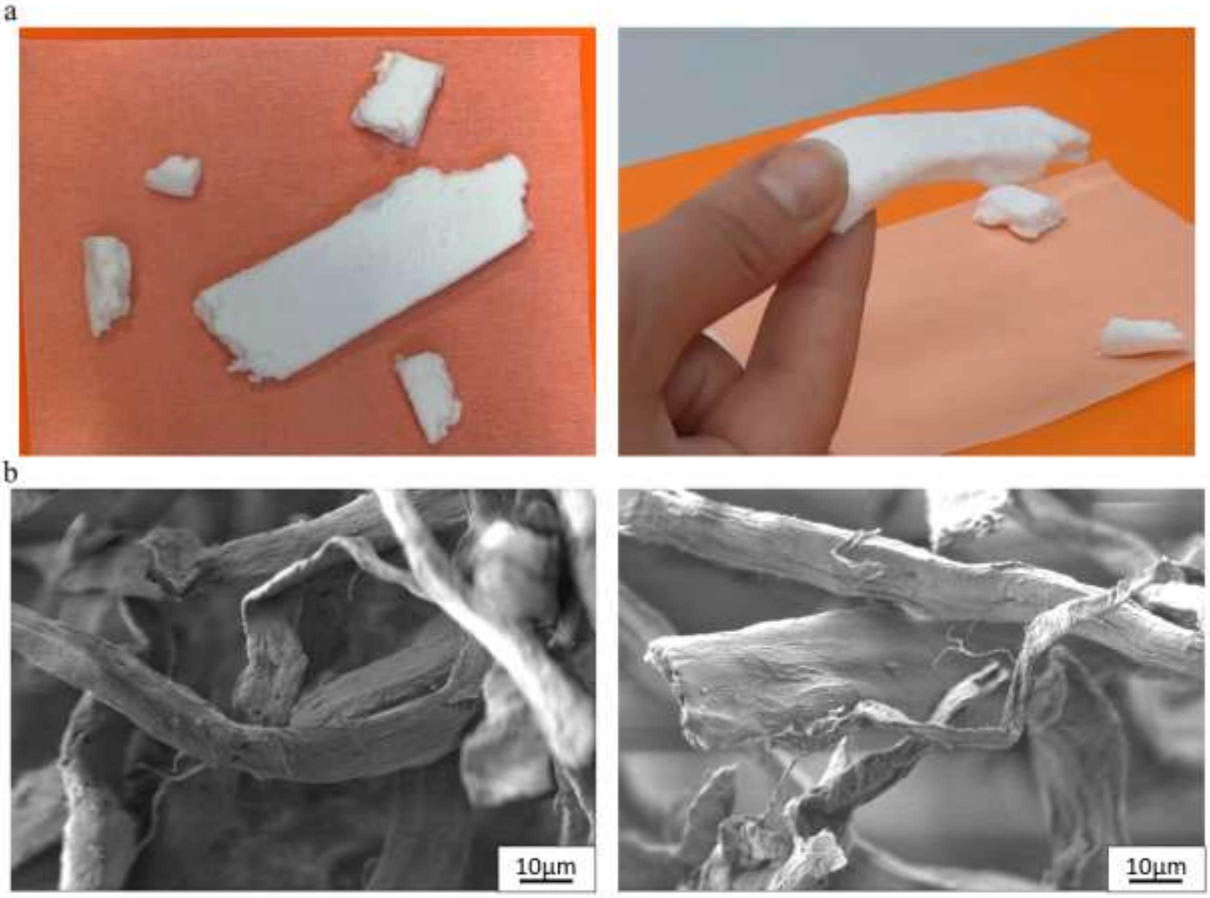

Figure S3. a) Digital photos and b) SEM images of freeze-dried cellulose fiber suspension.

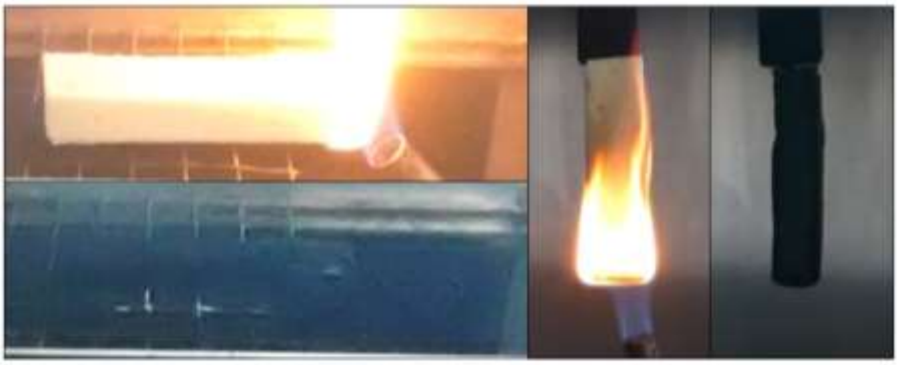

Figure S4. Horizontal and vertical flammability of commercial PU foam.

Table S1. Parameters derived from thermogravimetric analyses in nitrogen and air.

	Nitrogen			Air			
**Sample**	$T_{on,5\%}$ (°C)	T_{max} (°C)	Residue (%)	$T_{on,5\%}$ (°C)	T_{max1} (°C)	T_{max2} (°C)	Residue (%)
**1 BL**	260	305	22	261	295	496	<1
**2 BL**	254	291	30	250	285	493	<1
**3 BL**	248	286	33	246	279	533	1
**Cellulose**	289	352	10	287	332	465	<1

Table S2. Flammability test data.

	Horizontal		Vertical	
**Sample**	After-flame time (s)	Residue (%)	After-flame time (s)	Residue (%)
**1 BL**	26 ± 1	94 ± 1	37 ± 1	9 ± 1
**2 BL**	5 ± 3	98 ± 1	5 ± 1	89 ± 1
**3 BL**	<1	98 ± 1	<1	88 ± 1
**PU**	110 ± 10	30 ± 10	75 ± 5	40 ± 2

Table S3. Time to ignition (TTI), heat release rate (HRR), total heat release (THR) and total smoke production (TSR) from cone calorimetry.

Sample	TTI (s)	pkHRR (kW/m^2)	THR (MJ/m^2)	TSR (m^2/m^2)	Residue (%)
**Cellulose**	10 ± 1	167 ± 2	16.5 ± 0.4	1.2 ± 0.2	< 1
**1 BL**	9 ± 1	105 ± 7	13.9 ± 1.6	4.2 ± 3.0	5.5 ± 0.7
**2 BL**	9 ± 1	83 ± 1	6.9 ± 0.5	33.7 ± 14.1	16.1 ± 3.5
**3 BL**	9 ± 1	73 ± 1	4.2 ± 0.9	42.2 ± 18.9	22.9 ± 1.4
**PU**	9 ± 1	311 ± 24	31.0 ± 1.6	679.4 ± 37.8	20.9 ± 1.1

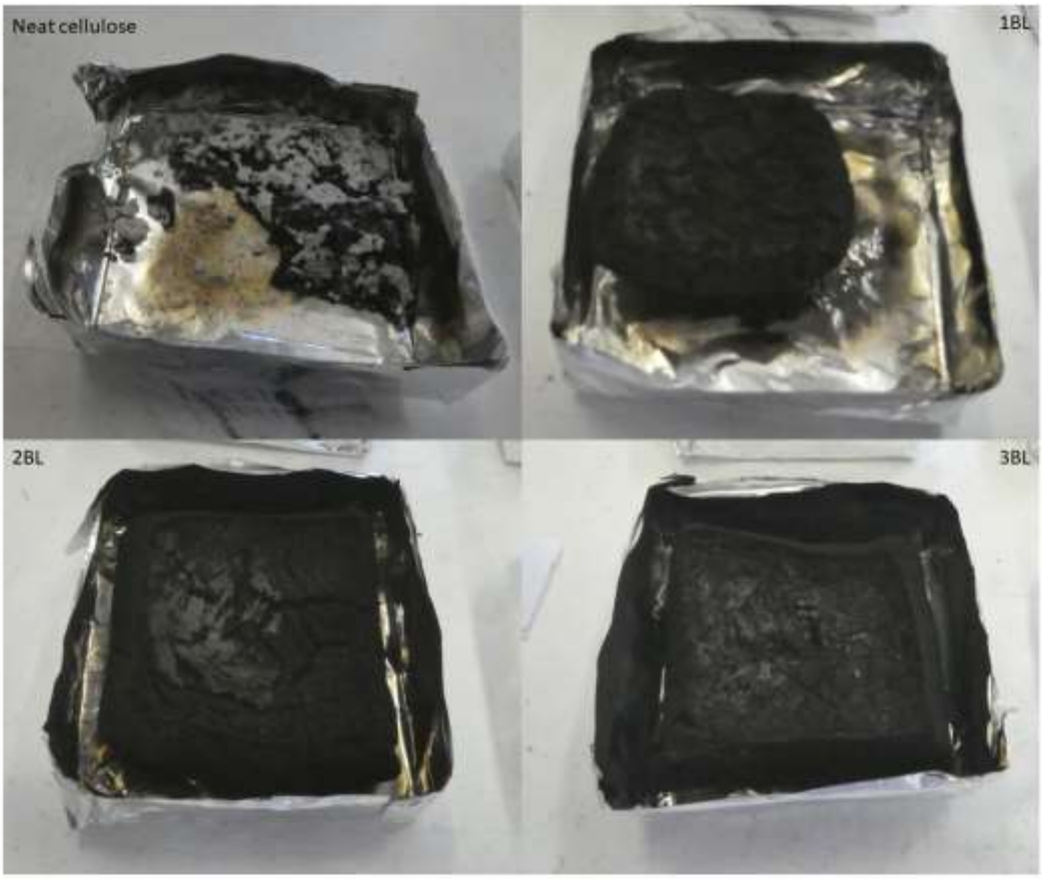

Figure S5. Digital photos of cone calorimetry residues.

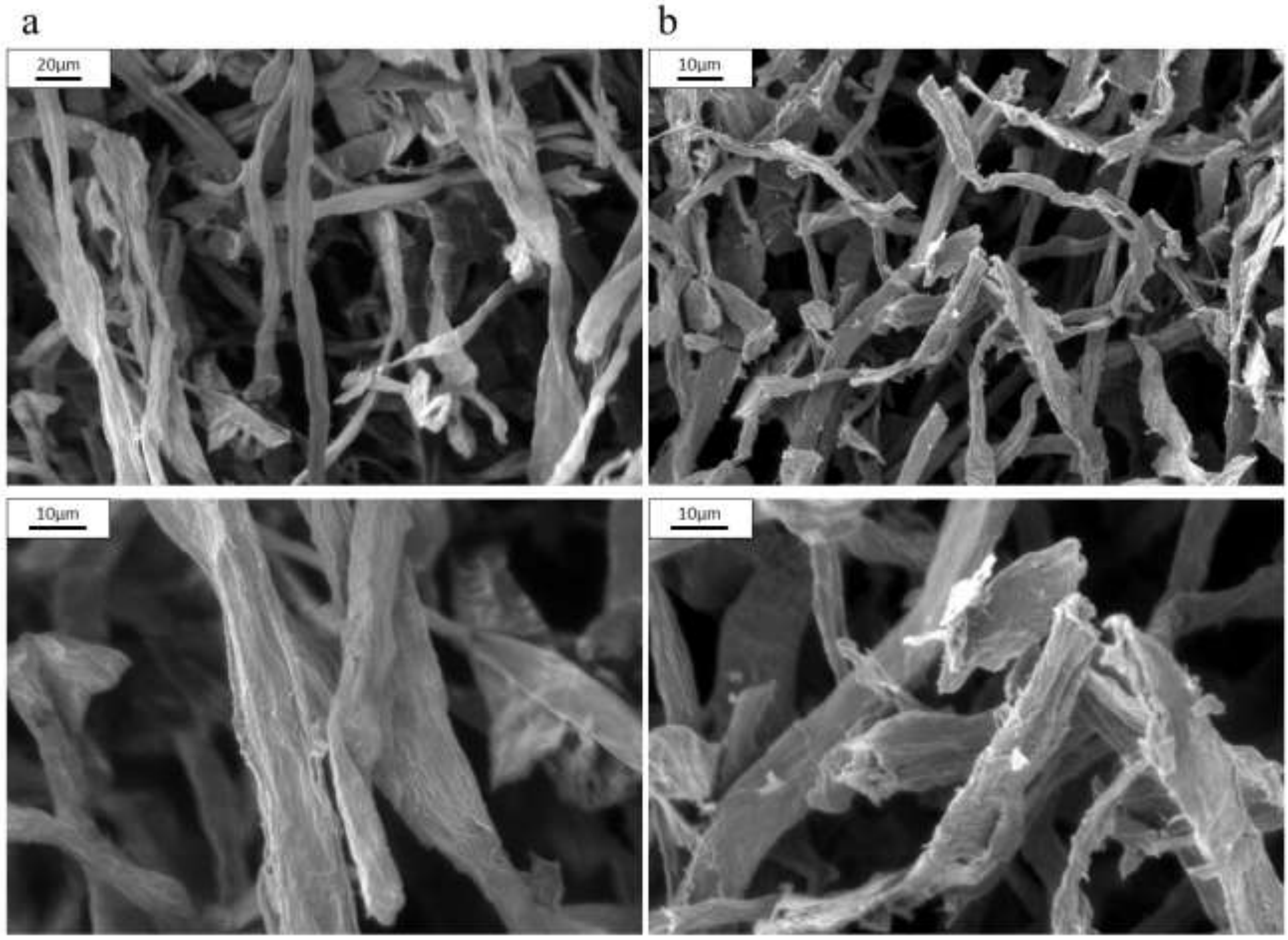

Figure S6. SEM images of the residue after cone calorimetry for fiber networks made by a) 1BL and b) 2BL coated fibers.

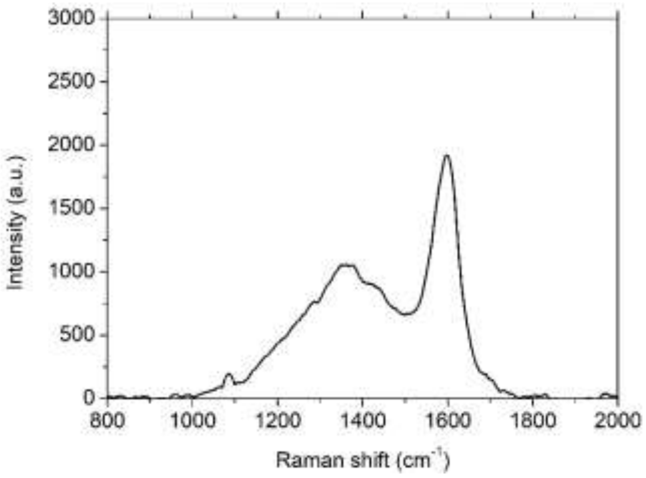

Figure S7. Raman spectrum of uncoated cellulose residue after cone calorimetry test.

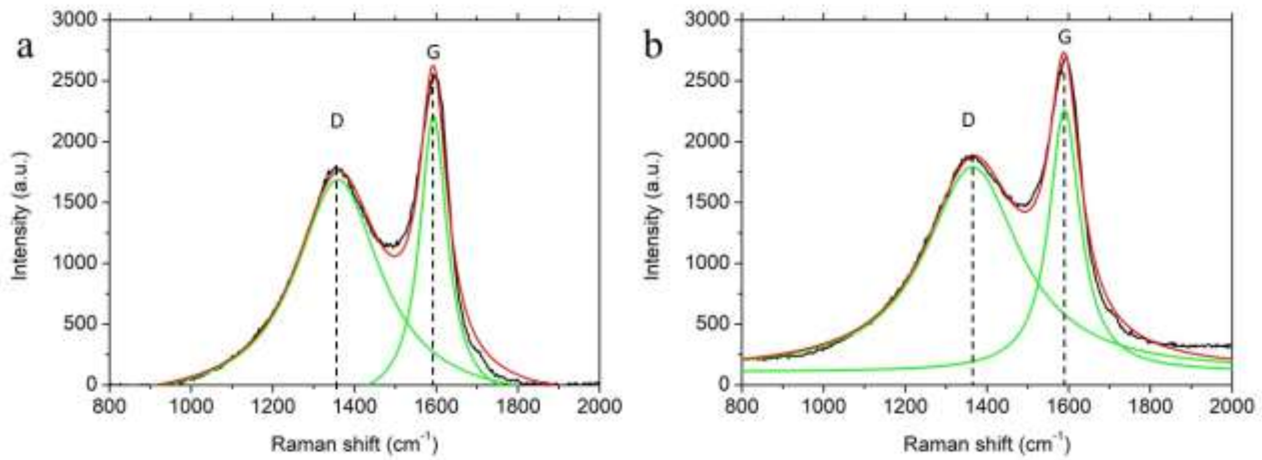

Figure S8. Raman spectra of a) 1BL and b) 2BL fiber network residues with deconvoluted fittings of peak D and G.
